# Migration from full‐head mask to “open‐face” mask for immobilization of patients with head and neck cancer

**DOI:** 10.1120/jacmp.v14i5.4400

**Published:** 2013-09-06

**Authors:** Guang Li, D. Michael Lovelock, James Mechalakos, Shyam Rao, Cesar Della‐Biancia, Howard Amols, Nancy Lee

**Affiliations:** ^1^ Department of Medical Physics Memorial Sloan‐Kettering Cancer Center New York NY USA; ^2^ Department of Radiation Oncology Memorial Sloan‐Kettering Cancer Center New York NY USA

**Keywords:** patient setup and immobilization, thermoplastic head mask, image‐guided radiotherapy, optical surface imaging, motion uncertainty

## Abstract

To provide an alternative device for immobilization of the head while easing claustrophobia and improving comfort, an “open‐face” thermoplastic mask was evaluated using video‐based optical surface imaging (OSI) and kilovoltage (kV) X‐ray radiography. A three‐point thermoplastic head mask with a precut opening and reinforced strips was developed. After molding, it provided sufficient visible facial area as the region of interest for OSI. Using real‐time OSI, the head motion of ten volunteers in the new mask was evaluated during mask locking and 15 minutes lying on the treatment couch. Using a nose mark with reference to room lasers, forced head movement in open‐face and full‐head masks (with a nose hole) was compared. Five patients with claustrophobia were immobilized with open‐face masks, set up using OSI and kV, and treated in 121 fractions, in which 61 fractions were monitored during treatment using real‐time OSI. With the open‐face mask, head motion was found to be 1.0 ± 0.6 mm and 0.4° ± 0.2° in volunteers during the experiment, and 0.8 ± 0.3 mm and 0.4° ± 0.2° in patients during treatment. These agree with patient motion calculated from pre‐/post‐treatment OSI and kV data using different anatomical landmarks. In volunteers, the head shift induced by mask‐locking was 2.3 ± 1.7 mm and 1.8° ± 0.6°, and the range of forced movements in the open‐face and full‐head masks were found to be similar. Most (80%) of the volunteers preferred the open‐face mask to the full‐head mask, while claustrophobic patients could only tolerate the open‐face mask. The open‐face mask is characterized for its immobilization capability and can immobilize patients sufficiently (< 2 mm) during radiotherapy. It provides a clinical solution to the immobilization of patients with head and neck (HN) cancer undergoing radiotherapy, and is particularly beneficial for claustrophobic patients. This new open‐face mask is readily adopted in radiotherapy clinic as a superior alternative to the standard full‐head mask.

PACS numbers: 87.19.xj, 87.63.L‐, 87.59.‐e, 87.55.tg, 87.55.‐x

## I. INTRODUCTION

Historically, the widely accepted standard of care for the immobilization of patients with head and neck (HN) cancer is the thermoplastic full‐head mask.^1^, ^2^, ^3^, ^4^ Closed‐form, full‐head masks have been well characterized in terms of head motion (~ 2 mm) within the mask^(^ ^2^ ^,^ ^5^ ^,^ ^6^ ^)^ and setup uncertainty (2–3 mm),^6^, ^7^, ^8^, ^9^ permitting the calculation of treatment margins to account for the geometric setup uncertainty of radiotherapy. However, the full‐head mask forces patients to keep the eyes and mouth closed during each daily treatment (a treatment period can last for more than 6 weeks). This is not only uncomfortable for many patients, but also intolerable for some, in particular those who suffer from claustrophobia. Although the full‐head mask can be cut open around the eyes and mouth to improve patients' comfort, mask cutting after molding is difficult, produces sharp edges, and has not been demonstrated to be helpful to claustrophobic patients. Therefore, there is a clinical demand for establishing a more practical alternative to the full‐head mask to improve patient comfort and tolerability while maintaining effectiveness.

Several methods have been reported to address this clinical need. Kim et al.^(10)^ developed a maskless technique that combines a customized head mold and a mouth‐bite tray with infrared reflectors to monitor motion. It was found that the immobilization system could be tolerated by claustrophobic patients with a ~ 2 mm range of motion, but it required eight repositioning procedures, on average, during 20 treatments. The investigators also applied a partial mask to immobilize the upper face and this cut down the number of repositioning procedures to two in 20 treatments. Due to oral toxicity, the biting effort on the mouthpiece and the change in treatment positions, there are concerns as to whether this system could reduce patient comfort and tolerability. In a study of 260 patients, Sharp et al.^(11)^ reported that a smaller full‐head mask, in comparison with the bigger head‐and‐shoulder mask, reduced claustrophobic anxiety in patients significantly, together with reduced skin toxicity,^(^ ^7^ ^,^ ^12^ ^)^ while the reproducibility of the setup and stability of the immobilization were not compromised. However, many patients still suffer from the enclosed sensation of the full‐head mask.

To characterize head motion within the mask, several methods have been applied, including the comparison of X‐ray images before and after treatment,^(^ ^1^ ^,^ ^9^ ^)^ and the use of infrared reflectors or emitters for motion monitoring with optical sensors^(13)^ or stereoscopic cameras.^(^ ^10^ ^,^ ^14^ ^)^ Recently, video‐based optical surface imaging (OSI) has been reported for monitoring head motion when a patient's facial area is visible in frameless and maskless stereotactic radiosurgery^(^ ^15^ ^,^ ^16^ ^)^ With a closed‐form, full‐head mask, this OSI method cannot be applied, since the full‐head mask blocks the facial area.

From our clinical observation, despite the same head alignment strategy being applied at simulation and treatment, a head rotation often appears in setup X‐ray images after the mask was placed and locked. Using open‐face mask and OSI, it allows us to monitor and quantify the head positioning change during mask‐locking process without ionization radiation. This piece of information is clinically useful in determining the uncertainty in patient setup, so as to the appropriate treatment margin in treatment planning.

In this study, we designed, characterized, and used an open‐face mask to immobilize patients with HN cancer and claustrophobic anxiety. We first discuss a volunteer study (N=10). Using the open‐face area as the region of interest (ROI), head motion was monitored using real‐time OSI motion monitoring during initial mask locking and for 15 minutes during which the volunteers were immobilized in the mask. The former quantifies head motion induced by mask locking for the first time, while the latter measures the absolute motion of the patient within the open‐face mask. Using a marked point on the nose in reference to the room lasers, the forced movement of volunteers within both open‐face and full‐head masks was compared. We then discuss the application of the open‐face mask in five patients with claustrophobia for treatments totaling 121 fractions. Both kilovoltage (kV) and OSI were applied for setup and assessment of patient motion. These results quantitatively characterize the immobilization performance of the open‐face mask.

## II. MATERIALS AND METHODS

### A. Precut open‐face mask and full‐head mask

**Figure 1:**
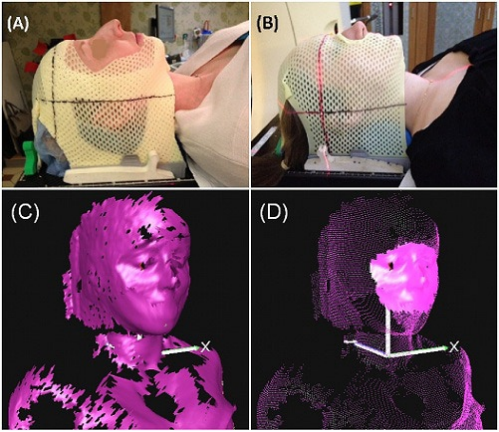
An open‐face mask and a conventional full‐head mask molded on two volunteers. An arbitrary alignment point was marked on the masks. For the open‐face mask (a), the open area was set to be the region of interest for AlignRT motion monitoring. For the conventional mask (b), the nose area was open, allowing alignment between a skin mark and the room laser in a forced motion test. A raw reference image (c), where the open area is clearly seen; the ROI (d) drawn on the reference image.

The open‐face mask is a three‐point (three locking points) thermoplastic head mask with a precut, isosceles‐trapezoid‐shaped opening, with reinforced strips around the opening and locking edges (Orfit Industries, Wijnegem, Belgium). After molding, the opening is enlarged to show the eyes, nose, mouth, and some forehead and cheek area. The patients are unable to see the mask. The opening, as shown in Fig. 1(a), was sufficiently large to serve as the ROI (Fig. 1(d)) using the OSI real‐time delta (RTD) mode for motion monitoring. The RTD mode provides three translational and three rotational motion shifts based on rapid auto‐registration of the real‐time images with the ROI of a reference image. The conventional three‐point full‐head mask has a small triangle‐shaped opening, which exposes the entire nose after being molded to the patient, as shown in Fig. 1(b). The immobilization performance of the conventional mask serves as a baseline for this study.

Both open‐face and conventional masks were prepared by immersion in a 65°‐70°C water bath for 5 minutes. During the molding process, the warm mask was centered on the nose. The superior and inferior edges were pressed against the forehead and chin, respectively, while the lateral opening edges were pressed against the cheek, as the mask was stretched and locked on the superior and lateral sides. A similar procedure was used for the conventional mask, with the small triangle‐shaped opening centered on the nose and stretched to show the entire nose. A standard head support, selected to fit the individual by minimizing the gap under the neck, was used for both open‐face and conventional masks. For patients, the simulation computed tomography (CT) scan was performed after the molding, whereas for volunteers, the mask was taken off after 10 minutes of cooling. All masks were used after 24 hours for the experiment or treatment.

### B. Optical Surface Imaging and Kilovoltage X‐ray Imaging

**Figure 2:**
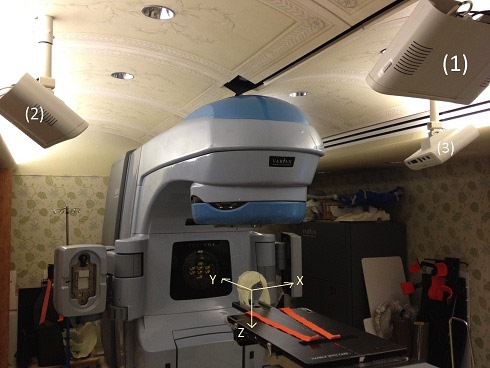
A video‐based optical surface imaging system (AlignRT) with three ceiling‐mounted stereoscopic camera pods in a treatment room with a linear accelerator (Trilogy).

A clinical OSI (AlignRT; VisionRT, London, UK) was used in this study. It contained three ceiling‐mounted stereoscopic camera pods in the treatment room of a linear accelerator (Trilogy; Varian Medical Systems, Palo Alto, CA), as shown in Fig. 2. The system was calibrated monthly and verified daily, using a calibration plate, which was placed at the machine isocenter. This system had two acquisition modes: static image capture (SIC) to acquire one image with high resolution, and RTD to continuously capture a series of images at a lower resolution. The frame rate was 2 to 3 frames per second (fps) in RTD mode, including image registration to the ROI at the mask opening. The ROI defined on CT‐based external contours was used for setup, whereas the ROI defined on an on‐site reference SIC image was used for RTD motion monitoring. In addition to RTD motion monitoring, head motion was also estimated based on pre‐ and post‐treatment SIC, using both CT‐based reference and on‐site SIC reference. When an on‐site SIC image was captured as the reference image, the accuracy of motion monitoring has 0.2 mm accuracy^(^ ^15^ ^,^ ^16^ ^)^ for head motion detection.

Using an on‐board imager (OBI; Varian Medical Systems, Palo Alto, CA), kV X‐ray imaging (orthogonal pair) was acquired for daily patient setup. Image registration was based on bony landmarks. The patient motion extracted from pre‐ and post‐treatment images was compared with the corresponding OSI data (SIC and RTD) for conformation using different anatomical landmarks. All 2D kV image registrations on bony landmarks were performed by therapists manually and verified by a physicist.

### C. Volunteers and patient selection

Ten volunteers (six male and four female) were accrued from employee workers in the Memorial Hospital. Both open‐face and full‐head masks were molded with a head support selected for each volunteer. An arbitrary isocenter was chosen and marked on the masks.

Five patients (three male and two female) who could not tolerate the conventional mask were selected to use the open‐face mask for immobilization during their simulation and multi‐fractionated treatment. Patients were simulated in a positron emission tomography (PET)/CT scanner (Discovery; GE Healthcare, Milwaukee, WI) for treatment planning. The disease sites were close to the facial area; they included the parotid, ear, neck, nasal skin, and base of tongue. For the patient with a disease site on the nasal skin, a bolus was taped on the skin in the opening area of the mask during his hypofractional treatment. The bolus and the rest of the opening surface were used as the ROI for RTD motion monitoring for this patient, after initial setup with the skin ROI in the opening area of his mask. Patient's Karnofsky performance status (KPS) ranged from 80 to 100.

### D. Experimental procedure for volunteers

Three experiments were performed to measure the following three motions for each volunteer: (1) mask‐locking‐induced head shifts; (2) head motion during 15 minutes of immobilization in the open‐face mask; and (3) forced movement inside both mask types.

*Mask‐locking‐induced head motion using RTD:* Each of the volunteers was aligned on the longitudinal midline along the nose and chin to the sagittal room laser, and on the tragus to the transverse (vertical) and coronal (horizontal) lasers. The open‐face mask was then loosely laid on the subject's face. A reference SIC image was acquired to serve as ideal setup position (since there is no CT image for volunteers) before each RTD acquisition during mask locking. The volunteers were informed that they should retain the head position and orientation despite the fact that they may sense their head being dragged away during mask locking. The procedure was repeated three times.

*Head motion during 15‐minute immobilization:* All volunteers were instructed to lie still for 15 minutes after a SIC image was captured as the reference for RTD.

**Figure 3:**
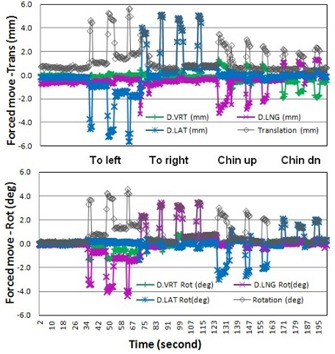
Demonstration of the forced motion in four directions of a volunteer subject wearing the open‐face mask. The colored lines are movements in vertical (D.VRT), longitudinal (D.LNG), and lateral (D.LAT) directions or rotational axes. The gray line is the motion amplitude. Note that after a forced motion, the subject's position tends to fall back to within 1.5 mm of its original baseline.

*Forced movement under the masks:* A dot was drawn with a marker pen on the tip of the subject's nose at the cross point of the sagittal and transverse lasers. The volunteer was instructed to move forcefully to the left and right, and to move chin up, and chin down. Using a ruler with an accuracy of 1.0 mm, the displacement between the skin mark and the laser line was measured, and three replicates were recorded in each direction. The absolute motions for left and right directions were averaged together, while chin‐up and chin‐down were averaged together. An example of RTD data for a volunteer with the open‐face mask is shown in Fig. 3. The absolute amplitudes of the motion in all four directions were calculated in both open‐face and full‐head masks for comparison.

### E. Clinical Procedure for Patients

All five patients were set up initially with OSI guidance. If the rotation of the head was found to be greater than 2° in any direction, the mask was unlocked, the patient was adjusted, and the mask was locked again to attempt to correct the head rotation. An orthogonal kV image pair was used for final setup. A SIC image was acquired after setup to serve as the reference for RTD and as the pretreatment OSI image. At the end of each treatment, both kV imaging and OSI were repeated to evaluate patient motions. This procedure was performed for all 121 fractions of treatment for the patients, except for RTD, which was performed in only 61 fractions. The RTD data were acquired throughout the entire treatment. Previously, a baseline drift was found in RTD acquisition due to possible thermal effect of the OSI system (0.5 mm drift in the vertical direction during the first 5–10 minutes), likely caused by the heat released from the central speckle projector.^(16)^ This drift was corrected by subtracting the baseline from RTD measurement curves.

## III. RESULTS

### A. Mask‐locking‐induced head shift in open‐face mask

Table 1 shows the RTD data during the initial mask locking process. On average, mask locking induces a head shift of 2.3 ± 1.7 mm and 1.8° ± 0.6°. It is interesting to note that the first locking almost always induces a higher head shift, and the shift levels off in the second and third locking trials. This result suggests that if a large shift of the head was induced in the first locking, a second trial is worthwhile to reduce it. In fact, we tried to correct head rotation in patients, if it was greater than 2°, with a second trial.

**Table 1:** Change in the head position of ten volunteers during the mask‐locking process using the open‐face mask and AlignRT motion monitoring. Three trials for each subject were performed. The first mask locking almost always caused greater head motion than the second and third trials. On average, a second trial in setup could reduce mask‐locking‐induced head shifts by 1 mm and 1°

*Volunteer*		*Translation (mm)*				*Rotation (°)*		
*Subject*	*Trial 1*	*Trial 2*	*Trial 3*	*Mean*	*Trial 1*	*Trial 2*	*Trial 3*	*Mean*
1	0.1	0.3	0.9	0.4	1.9	1.9	1.5	1.8
2	4.5	1.8	2.2	2.8	4.0	1.9	1.8	2.6
3	3.5	2.9	3.0	3.2	1.6	0.8	0.8	1.0
4	3.0	2.0	1.2	2.1	2.5	1.3	2.6	2.1
5	2.6	0.5	0.5	1.2	3.0	1.4	2.0	2.1
6	2.2	0.5	1.0	1.2	2.1	0.8	1.4	1.4
7	4.3	3.1	2.6	3.3	1.3	1.4	1.2	1.3
8	1.4	1.2	0.3	1.0	1.0	1.3	0.8	1.0
9	9.9	4.6	4.3	6.3	4.2	1.8	2.1	2.7
10	2.2	1.9	1.4	1.9	3.1	1.0	1.2	1.8
Average	3.4	1.9	1.8	2.3	2.5	1.4	1.5	1.8
St dev	2.7	1.4	1.3	1.7	1.1	0.4	0.6	0.6

### B. Comparison of Forced Movement in Open‐Face and Full‐Head Masks

Table 2 shows the comparison of the forced (translational) movement of ten volunteers in both masks. On average, the open‐face mask allows a maximum movement (from left or right and chin up or chin down actions) of 5.5 ± 2.5 mm (lateral) and 4.2 ± 2.0 mm (longitudinal). Eight out of ten subjects in this study preferred the open‐face mask to the full‐head mask, based on their comfort level during the experiment. Figure 3 shows RTD motion curves (3–4 replicates for each direction) in six degrees of freedom, including rotational shifts. Also note that for chin‐up and chin‐down moves, vertical position was also changed, but not recorded in Table 2. This RTD test cannot be performed for the full‐head mask, as the nasal surface is too small to produce any reliable result. An important phenomenon observed in all volunteers is that no matter how the forced motion occurs, when the force is released, the head will fall back to its original position within 1 to 2 mm, as shown in Fig. 3.

**Table 2:** Comparison of the forced motion of volunteers wearing the open‐face mask or conventional full‐head mask. Six measurements (three in each direction) were performed to calculate the mean motion using a skin marker against the room laser reference. On average, the open‐face mask allowed a larger maximum shift than the conventional mask by 1 mm. This was because the edges of the nose opening in the conventional mask caused the volunteers discomfort when they were asked to move in some directions

	*Left or Right (mm)*				*Chin Up or Down (mm)*				
	*Open‐face mask*		*Full‐head mask*		*Open‐face mask*		*Full‐head mask*		*Preference*
*Volunteer*	*Mean*	*St dev*	*Mean*	*St dev*	*Mean St dev*	*Mean*	*St dev*	*(OF vs. FH)*
1	7.3	0.5	6.3	1.2	7.3	1.5	6.3	3.0	OF
2	2.8	0.4	2.7	0.8	1.9	1.0	2.0	0.0	OF
3	2.6	0.5	1.9	0.2	2.3	0.4	2.3	0.5	OF
4	7.9	0.7	2.9	0.7	6.2	0.8	3.7	0.5	OF
5	7.9	1.5	4.0	1.1	5.3	3.3	2.0	0.0	OF
6	7.6	1.6	3.5	1.2	4.2	0.8	3.3	1.5	FH
7	3.3	0.5	1.0	0.0	3.7	0.5	2.8	0.8	OF
8	2.3	1.1	1.7	0.5	1.8	0.8	1.8	0.4	OF
9	8.1	1.3	8.8	1.0	6.5	0.6	3.0	0.6	FH
10	5.0	1.9	9.5	1.4	2.7	1.4	5.2	2.3	OF
Average	5.5	1.0	4.2	0.8	4.2	1.1	3.3	1.0	8OF vs. 2FH
St dev	2.5		3.0		2.0			1.5	

OF = open‐face mask; FH = full‐head mask.

### C. Head immobilization and stability using open‐face mask

**Figure 4:**
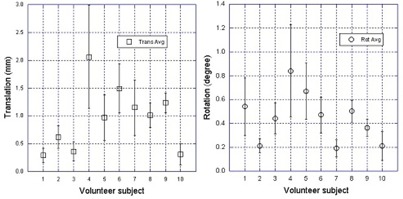
Translational and rotational motion (vector) average with standard deviation (error bar) in 15 minutes for ten volunteers. The mean is 1.0 ± 0.6 mm and 0.4° ± 0.4°.

**Figure 5:**
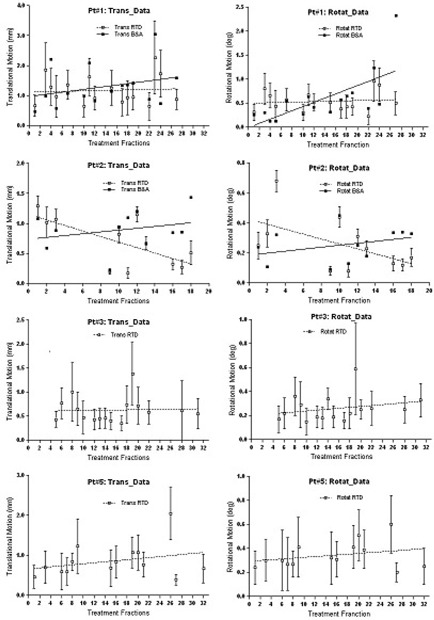
The head motion (vector) of four patients during radiation treatment in 11 to 16 fractions per patient. The mean for all patients and fractions is 1.5 mm and 0.5°, which is consistent with the volunteer data shown in Fig. 4. Although patient weight loss was observed as the mask fit more loosely in later fractions, no significant increase in motion was observed. The linear regression fit (dotted line) is shown in each graph. In the first 2 patients, the shift difference between before and after (B&A) SIC images is provided.

Figure 4 shows the motion of the heads of ten volunteers in the open‐face mask, which averaged 1.0 ± 0.6 mm and 0.4° ± 0.4°. During the 15 minutes of immobilization, the volunteers blinked their eyes frequently, but this had no noticeable effect (< 1 mm) on the motion of their heads. Figure 5 shows the RTD data from four patients during 57 fractions of treatment. The average motion was found to be 0.8 ± 0.3 mm and 0.4° ± 0.2°. This is in a close agreement with the volunteer data discussed above. It is interesting to note that although the mask becomes loose in later fractions, head motion remains around the same level.

### D. Confirmation of intrafractional motion with pre‐/post‐treatment kV and OSI data

Table 3 shows the comparison between the patients' RTD and pre‐ and post‐treatment SIC data using OSI. The translational data from pre‐ and post‐treatment kV is also included. The RTD data are in close agreement with the OSI results using the on‐site ROI as the reference image, but differs from OSI results using the CT‐based ROI as the reference image, mostly due to the rotational difference between the simulation position and treatment position. The kV data fall in between OSI‐1 and OSI‐2, since the alignment of kV images tends to compromise among different bony landmarks in the presence of head rotation.

**Table 3:** Head motion of five claustrophobic patients monitored with AlignRT in real time during treatment, and with both AlignRT and orthogonal kV imaging in pre‐ and post‐treatment (P&P). The average motion of these patients is similar to that of the volunteers. The X‐ray P&P data fall between two sets of AlignRT P&P data (OSI‐1 and OSI‐2) using different reference images. Note: both OSI‐1 and OSI‐2 show P&P differences, quantifying head motion during treatment, although OSI‐1 is more reliable since it does not carry residual head rotation at setup

				*Translation (mm)*					*Rotation (degree)*			
				*RTD*		*P&P X‐ray*			*RTD*		*P&P*	
*Patient*	*Sex*	*Age*	*fx/Fx^a^*	*Mean*	*St dev*	*OSI‐1^b^*	*OSI‐2^c^*	*kV*	*Mean*	*St dev*	*OSI‐1*	*OSI‐2*
1	M	65	16/30	1.2	0.6	1.3	2.3	1.7	0.5	0.2	0.6	1.1
2	F	72	11/20	0.7	0.1	0.9	1.5	1.0	0.3	0.1	0.3	1.2
3	M	59	16/33	0.6	0.3	0.5	1.4	1.2	0.3	0.1	0.2	0.6
4	M	76	4/5	0.5	0.2	1.3	1.0	1.1	0.6	0.2	0.8	0.2
5	F	55	14/33	0.9	0.4	1.1	1.6	1.4	0.4	0.2	0.4	0.8
*Average*		65	12/24	0.8	0.3	1.0	1.6	1.4	0.4	0.2	0.5	0.8
*St dev*				0.3		0.4	0.5	0.5	0.2		0.3	0.4

a fx/Fx refers to the number of fractions (fx) for which AlignRT was applied for motion monitoring over the total number of treatment fractions (Fx).

b OSI‐1 uses the on‐site AlignRT image as the registration reference image, for pre‐ and post‐treatment registration.

c OSI‐2 uses the CT external contours as the registration reference image, for pre‐ and post‐treatment registration.

## IV. DISCUSSION

### A. Absolute head motion inside open‐face mask

In this study, we measured head stability in 15 human subjects inside the open‐face mask during 15 minutes or more using RTD. Head stability was confirmed by kV X‐ray imaging in patients, providing direct and quantitative results on the quality of the new open‐face mask in the clinic. This RTD measurement cannot be conducted with the conventional enclosed full‐head mask, since the ROI on the nose is insufficient for monitoring. The ROI, which is defined as the entire opening area of the mask (Fig. 1), covers most of the central face area, which was reported useful for phantom and patient setup,^(17)^ although a smaller ROI tends to result in nosier registration results. Previously, surface or marker imaging over the mask itself has been reported as a surrogate for patient motion,^(^ ^18^ ^,^ ^19^ ^)^ but we consider direct imaging of the skin more accurate and reliable. For instance, if full‐head mask becomes lose, then small head motion within the conventional mask may not be detectable. Furthermore, the uneven surface of the mask introduces uncertainty in surface imaging and registration. In this study, although relative comparison of the full‐head mask is limited to only point‐checking in forced movement, the information on absolute motion for the open‐face mask, together with setup uncertainty, is useful in the determination of treatment margins for this immobilization method.

From the forced movement experiment, it is interesting to see that, although the reinforcing strips in the open‐face mask (Fig. 1) makes it sturdier than the conventional mask, subjects can move slightly more in the open‐face masks than in the full‐head masks. In the conventional mask, the presence of the sharp edge around the nose opening limits further movement when it touches the nose. According to the manufacturer (Orfit Industries), the strength of the two masks in resistance to an external horizontal force is almost identical in their head phantom tests. This is consistent with our finding in this study: the maximum range of the voluntary motion for both masks is similar, around 10 mm. On the other hand, the immobilization device is designed primarily to prevent involuntary head motion, which is much smaller in scale, as shown in Table 3, Fig. 4, and Fig. 5. Clinically, the forced movements rarely happen, but the experiment provides the limits on extreme head movements (as shown in Table 2) and likelihood of settling back to the original position after relaxation (as shown in Fig. 3). The gating function of AlignRT in RTD mode can be used to turn the treatment beam off, in case patients did move substantially within the open‐face mask. In contrast, this real‐time imaging option is not available for full‐head mask. Although a mouth‐bite infrared reflector array could be used for optical motion tracking, this method is not ideal for HN cancer patients due to potential oral toxicity.

In comparison with previously published data on the full‐head mask,^(^ ^2^ ^,^ ^5^ ^,^ ^6^ ^)^ our data on head motion inside the open‐face mask show consistent results for both volunteers and patients. Any involuntary head motion is restricted to within 2 mm at 95% confidence level. Note that the average age is around 35 for volunteers and 65 for patients. In addition, because of the facial opening in the mask, we can also evaluate the shift in head position induced by mask‐locking. This observation provides direct quantified evidence of mask‐locking‐induced head motion for the first time, and further evaluation may be needed, including additional evaluation on different days and by different users. One outlier with ~ 1 cm dragged distance could have resulted from several factors: the female subject's hair got in the way of the locking slot, her mask appeared to be extremely tight, and she did not apply enough counter‐dragging force to retain head position. More importantly, the result derived from the open‐face mask could apply to the full‐head mask where real‐time motion detection on patient skin is not possible. Our study has shown that this open‐face mask can be used in routine radiotherapy, providing immobilization within 2.0 mm, with improved patient comfort and tolerability.

### B. General concerns for patient comfort

This study has demonstrated that some patients, who suffer from claustrophobic anxiety and cannot tolerate the conventional full‐head mask, can, in fact, tolerate the open‐face mask. This suggests that the open‐face mask can be applied with a larger population of patients with improved comfort. It has been reported that patients who are covered less experience less claustrophobic distress while being immobilized. Sharp et al.^(11)^ reported that use of a smaller three‐point full‐head mask reduced claustrophobic feelings and skin toxicity, in comparison with a larger five‐point head‐and‐shoulder mask, while similar patient immobilization was retained. Kim et al.^(10)^ reported that an upper‐face mask without chin restriction can ease patient claustrophobic anxiety, while a mouth‐bite tray can be used to place infrared reflectors to monitor head motion. Severely claustrophobic patients, however, may not be able to tolerate the open‐face mask. In this case, daily prior‐treatment medication, including anesthesia, may have to be used for the entire treatment period. With mild or moderate claustrophobic patients, the open‐face mask has performed well in our radiotherapy clinic.

We surveyed the volunteers about the level of comfort they experienced in both masks immediately after they wore them in the experiment. Eight out of the ten volunteers preferred the open‐face mask. With the full‐head mask, subjects are forced to keep their eyes and mouth closed, leaving them in the “dark” during the 20‐minute treatment everyday for six weeks. The open‐face mask removes this restriction. This advantage, along with those discussed above, makes the use of the open‐face mask favorable over the conventional full‐head mask in the clinic.

### C. Other Clinical Considerations

It is also worthwhile to note that the size of the facial opening is critical to both the quality of immobilization and visualization of OSI (SIC and RTD). There is a tradeoff between them, and we found that the size of the opening of the open‐face mask can achieve clinically accept‐able levels for both immobilization and surface imaging. We consider that the ROI meets the minimal requirement for an accurate and reliable registration for setup and motion monitoring. Because of the small ROI at the opening (Fig. 1(d)), a change in facial expression, such as smiling or squeezing the nose, could cause an apparent shift in RTD signal or a false positive. However, a patient on treatment is unlikely to have these facial expressions, and even if they do, the motion signal will fall back to the original level after the patient has relaxed. For other minor facial expressions, such as blinking, we found that they did not cause a noticeable change (< 1 mm) in RTD motion monitoring.

Due to the opening in the open‐face mask, the cast line on the mask may not form a cross point for setup. Therefore, the alignment of the patient with the room lasers has to rely on an incomplete line. In some cases, the open‐face mask could be twisted during the six‐week treatment course, resulting in two partial lines that no longer align with the laser line. This could result from changes in patient anatomy during the course of treatment, as we have observed the looseness of the mask in later fractions. In this case, we recommend that the difference is split for conventional setup.

We are currently investigating the accuracy of patient setup using daily kV imaging and comparing the open‐face and full‐head masks in patients. The collection of setup 2D kV images from two cohorts of patients is completed and analyzed: one with open‐face masks and the other with full‐head masks, aiming to make a direct comparison of the two masks. A followup report is expected on using different bony landmarks for registration between 2D kV and digitally reconstructed radiograph of planning CT for setup evaluation and between pre‐ and post‐treatment 2D kV images for motion assessment.

We have used OSI to quantify head motion during 15–20 minute period for both volunteers and HN patients with open‐face mask. On HN patients, the motion range was confirmed with pre‐ and post‐treatment 2D kV and OSI static imaging. The absolute motion range was measured to assess motion uncertainty, which can be incorporated in the margin for planning tumor volume using this immobilization system. In addition, mask‐locking‐induced motion was measured using real‐time OSI, and further investigation is worthwhile to assess this uncertainty systematically for conventional patient setup. Although a large head movement rarely occurs during treatment, once it happens as simulated in forced movement experiments, upon relaxation the head almost always settles back to its original position within 2 mm.

## V. CONCLUSIONS

We have established and characterized a new precut open‐face thermoplastic mask for immobilization of patients with head and neck, cervical spine, and brain cancer during radiotherapy. Using real‐time OSI, absolute head motion during 15 minutes or more on the treatment couch was found to be approximately 1.0 ± 0.5 mm for both healthy volunteers and patients with claustrophobia. Mask‐locking‐induced head shifts and rotations were quantified and accounted for as the uncertainty in conventional setup. The open‐face mask opening (with reinforced strips) does not reduce the strength of the mask, in comparison with the full‐head mask. The new open‐face mask improves comfort and tolerability for patients, particularly those with moderate claustrophobic anxiety, and can be readily applied in clinical practice.

## ACKNOWLEDGMENTS

The authors thank Mr. Marty Ratner (Orfit Industries) and Dr. Norman Smith (VisionRT Inc.) for their cooperation and for manufacturing the designed open‐face masks for this clinical study. We sincerely thank all participating therapists at CT simulation and treatment (R443), especially Ms. Marcia Chong Ton and Ms. Laura Boone, for their participation and dedication to high‐quality patient care. Finally, we are grateful to all the volunteers in this study, including ZS, RK, BS, SR, ML, YL, FL, LF, JT, and KE. We appreciate the comments from the peer‐review process, which have invoked improvements for this manuscript.

Appendix A: Example of how team members may work together and in parallel during the process.

Siegelman JRQW., Gress DA. Radiology Stewardship and Quality Improvement: The Process and Costs of Implementing a CT Radiation Dose Optimization Committee, in a Medium Sized Community Hospital System. In press, J Am Coll Radiol (accepted December 2012).

## References

[1] Verhey LJ , Goitein M , McNulty P , Munzenrider JE , Suit HD . Precise positioning of patients for radiation therapy. Int J Radiat Oncol Biol Phys. 1982;8(2):289–94. 6282792 10.1016/0360-3016(82)90530-2

[2] Thornton AF, Jr. , Ten Haken RK , Gerhardsson A , Correll M . Three‐dimensional motion analysis of an improved head immobilization system for simulation, CT, MRI, and PET imaging. Radiother Oncol. 1991;20(4):224–28. 2068339 10.1016/0167-8140(91)90120-6

[3] Willner J , Flentje M , Bratengeier K . CT simulation in stereotactic brain radiotherapy ‐ analysis of isocenter reproducibility with mask fixation. Radiother Oncol. 1997;45(1):83–88. 9364636 10.1016/s0167-8140(97)00135-7

[4] Gilbeau L , Octave‐Prignot M , Loncol T , Renard L , Scalliet P , Gregoire V . Comparison of setup accuracy of three different thermoplastic masks for the treatment of brain and head and neck tumors. Radiother Oncol. 2001;58(2):155–62. 11166866 10.1016/s0167-8140(00)00280-2

[5] Tsai JS , Engler MJ , Ling MN , et al. A non‐invasive immobilization system and related quality assurance for dynamic intensity modulated radiation therapy of intracranial and head and neck disease. Int J Radiat Oncol Biol Phys. 1999;43(2):455–67. 10030275 10.1016/s0360-3016(98)00398-8

[6] Tryggestad E , Christian M , Ford E , et al. Inter‐ and intrafraction patient positioning uncertainties for intracranial radiotherapy: a study of four frameless, thermoplastic mask‐based immobilization strategies using daily cone‐beam CT. Int J Radiat Oncol Biol Phys. 2011;80(1):281–90. 20951506 10.1016/j.ijrobp.2010.06.022

[7] Velec M , Waldron JN , O'Sullivan B , et al. Cone‐beam CT assessment of interfraction and intrafraction setup error of two head‐and‐neck cancer thermoplastic masks. Int J Radiat Oncol Biol Phys. 2010;76(3):949–55. 20056344 10.1016/j.ijrobp.2009.07.004

[8] Kang H , Lovelock DM , Yorke ED , Kriminski S , Lee N , Amols HI . Accurate positioning for head and neck cancer patients using 2D and 3D image guidance. J Appl Clin Med Phys. 2011;12(1):3270. 10.1120/jacmp.v12i1.3270 PMC3065820 21330971

[9] Ohtakara K , Hayashi S , Tanaka H , et al. Clinical comparison of positional accuracy and stability between dedicated versus conventional masks for immobilization in cranial stereotactic radiotherapy using 6‐degree‐of‐freedom image guidance system‐integrated platform. Radiother Oncol. 2012;102(2):198–205. 22100656 10.1016/j.radonc.2011.10.012

[10] Kim S , Akpati HC , Li JG , Liu CR , Amdur RJ , Palta JR . An immobilization system for claustrophobic patients in head‐and‐neck intensity‐modulated radiation therapy. Int J Radiat Oncol Biol Phys. 2004;59(5):1531–39. 15275741 10.1016/j.ijrobp.2004.01.025

[11] Sharp L , Lewin F , Johansson H , Payne D , Gerhardsson A , Rufgvist LE . Randomized trial on two types of thermoplastic masks for patient immobilization during radiation therapy for head‐and‐neck cancer. Int J Radiat Oncol Biol Phys. 2005;61(1):250–56. 15629618 10.1016/j.ijrobp.2004.04.047

[12] Lee N , Chuang C , Quivey JM , et al. Skin toxicity due to intensity‐modulated radiotherapy for head‐and‐neck carcinoma. Int J Radiat Oncol Biol Phys. 2002;53(3):630–37. 12062606 10.1016/s0360-3016(02)02756-6

[13] Schulte RW , Fargo RA , Meinass HJ , Slater JD , Slater JM . Analysis of head motion prior to and during proton beam therapy. Int J Radiat Oncol Biol Phys. 2000;47(4):1105–10. 10863084 10.1016/s0360-3016(00)00551-4

[14] Ryken TC , Meeks SL , Pennington EC , et al. Initial clinical experience with frameless stereotactic radiosurgery: analysis of accuracy and feasibility. Int J Radiat Oncol Biol Phys. 2001;51(4):1152–58. 11704340 10.1016/s0360-3016(01)01756-4

[15] Cervino LI , Pawlicki T , Lawson JD , Jiang SB . Frame‐less and mask‐less cranial stereotactic radiosurgery: a feasibility study. Phys Med Biol. 2010;55(7):1863–73. 20224158 10.1088/0031-9155/55/7/005

[16] Li G , Ballangrud A , Kuo LC , et al. Motion monitoring for cranial frameless stereotactic radiosurgery using video‐based three‐dimensional optical surface imaging. Med Phys. 2011;38(7):3981–94. 21858995 10.1118/1.3596526

[17] Peng JL , Kahler D , Li JG , et al. Characterization of a real‐time surface image‐guided stereotactic positioning system. Med Phys. 2010;37(10):5421–33. 21089778 10.1118/1.3483783

[18] Li S , Liu D , Yin G , Zhuang P , Geng J . Real‐time 3D‐surface‐guided head refixation useful for fractionated stereotactic radiotherapy. Med Phys. 2006;33(2):492–503. 16532957 10.1118/1.2150778

[19] Linthout N , Verellen D , Tournel K , Storme G . Six dimensional analysis with daily stereoscopic x‐ray imaging of intrafraction patient motion in head and neck treatments using five points fixation masks. Med Phys. 2006;33(2):504–13. 16532958 10.1118/1.2165417

